# Cervical cancer screening utilization among healthcare professionals in Ethiopia: systematic review and meta-analysis

**DOI:** 10.3389/fgwh.2025.1467313

**Published:** 2025-02-18

**Authors:** Michael Amera Tizazu, Addisalem Workie Demsash, Tadesse Mamo, Tirusew Nigussie Kebede, Abebe Mihretie, Kassa Mamo Negash, Fetene Kassahun Amogne, Abate Dargie Wubetu

**Affiliations:** ^1^ School of Public Health, Asrat Woldeyes Health Science Campus, Debre Berhan University, Debre Berhan, Ethiopia; ^2^ Department of Health Informatics, Asrat Woldeyes Health Science Campus, Debre Berhan University, Debre Berhan, Ethiopia; ^3^ Department of Midwifery, Asrat Woldeyes Health Science Campus, Debre Berhan University, Debre Berhan, Ethiopia; ^4^ Department of Psychiatry, Asrat Woldeyes Health Science Campus, Debre Berhan University, Debre Berhan, Ethiopia

**Keywords:** cervical cancer screening, utilization, healthcare professionals, systematic review, meta-analysis, Ethiopian

## Abstract

**Background:**

Cancer of the cervix is the second most common cancer among women worldwide, with about over 660 000 new cases and approximately ninety-four percent of the 350 000 cervical cancer-related death happened in low- and middle-income countries. Effective screening initiatives are particularly crucial in preventing cervical cancer in women. Therefore, the purpose of this systematic literature review was to investigate the pooled prevalence of Ethiopian female healthcare professionals' cervical cancer screening utilization.

**Methods:**

Published articles were searched from different major international databases (PubMed, Cochrane Library, Scopus, Web of Science, Since Direct, Google Scholar). Direct Google searches were used for additional sources mainly for gray and preprint studies. This review included studies that reported either the use of cervical cancer screening or cervical cancer screening predictors in Ethiopia. All published and unpublished studies through May/2024 and reported in the English language were retrieved to assess eligibility for inclusion in this review. The Newcastle-Ottawa Scale quality assessment tool was used to assess the quality of the included studies and Egger's test was used to assess the publication bias.

**Results:**

In order to calculate the pooled prevalence of cervical cancer screening, 2,919 female healthcare professionals participated in the review. Articles were published from 2015 to 2024. The pooled Utilization of cervical cancer screening in Ethiopia, as determined by a meta-analysis of ten articles was 13.59% (95% CI: 7.53, 19.65).

**Conclusion and recommendation:**

The estimated/pooled cervical cancer screening utilization was found to be lower than the World Health Organization recommendations as the estimator revealed in the meta-analysis. The low utilization of Cervical Cancer (CCa)screening practice despite they are healthcare professionals is a significant concern that can impact the broader efforts to combat cervical cancer. Based on the this reviews the authors recommend regular monitoring and evaluation of the CCa screening habits of healthcare professionals and the effectiveness of implemented interventions. It is necessary to explore the factors that enable or hinder CCa screening and address the issue through qualitative or mixed-method studies.

## Introduction

Cervical Cancer(CCa) is the second most common cancer among women worldwide, with over 660 000 new cases and approximately ninety-four percent of the 350 000 cervical cancer-related death happened in low- and middle-income nations (1, 2). In low- and middle-income countries (LMICs), including Ethiopia, cervical cancer is the commonest cancer affecting reproductive organs and also the leading cause of death from cancer among women. Studies estimated that 20.9 million women were at risk of developing cervical cancer in Ethiopia with an estimated 4,648 and 3,235 annual numbers of new cases and deaths, respectively (2, 3).

Comprehensive cervical cancer control methods, such as primary prevention (HPV vaccination), secondary prevention (screening and treatment of pre-cancerous lesions), tertiary prevention (diagnosis and treatment of invasive cervical cancer), and palliative care, can therefore be used to prevent it (4, 5). These precautions haven't, however, been applied consistently throughout and among nations. Only forty-four percent of women in LMICs were screened for CCa, with Sub-Saharan African women having the lowest prevalence (5). In high-income countries, the percentage of women who were screened for the disease is greater than 60%. While in Ethiopia the proportion of CCa screening was very low (15.79%) as reported in 2021 (6, 7).

Effective screening initiatives are particularly crucial in preventing CCa in women who have not had a vaccination (7). In conjunction with prompt and effective treatment of precancerous lesions, the World Health Organization (WHO) recommends screening women aged 30–49 years through visual inspection with acetic acid (VIA) in low-resource settings, a Papanicolaou test (cervical cytology) every 3–5 years, or HPV testing every 5 years (7, 8).

Ethiopia's first cancer prevention and control strategy was released in 2015 (9). VIA and cryotherapy, which were determined to be practical and suitable screening and treatment modalities, have been used in a “screen-and-treat” approach (10). CCa screening utilization is still low (11–15) even after this approach has been put into practice. This can be attributed to a number of factors, including low knowledge (16), negative attitude (17), inadequate diagnostic facilities (18), an unstructured referral system (19), poor infrastructure (20), and being young women (21). Women belonging to the lower social class, life style practiced by women such as sexual and hygienic behavior, increased parity, smoking, early initiation of sexual intercourse, multiple sexual partners among couples, that can expose to Human papilloma virus (HPV) which is a major cause of cervical cancer have been identified as a risk factor in other studies (22–24). HPV with different variants like type 16, 18, 31, 33, and 45 are mostly related with invasive carcinoma of the cervix. Thus, infection with HPV will alter the nature of cervical cell that can be detected early if there is adequate screening technique (24, 25).

The crucial issue is that all individuals and couples should have access to comprehensive, high-quality reproductive healthcare and services, including the prevention of CCa, and that using these services is essential to the socioeconomic advancement and general well-being of all Ethiopian citizens, particularly women. If a woman has CCa and is diagnosed and treated promptly, screening can save her life. In order to save lives and lessen the suffering that many women with CCa endure, it is essential that all medical professionals to advocate for this service. Therefore, the purpose of this systematic literature review was to investigate Ethiopian female healthcare professionals' use of cervical cancer screening.

## Methods

### Data sources and search strategies

The aim of this systematic literature review and meta-analysis was to estimate the pooled uptake of CCa screening utilization among female healthcare professionals in Ethiopia. The findings of this review have been reported according to the recommendation of the Preferred Reporting Items for Systematic Review and Meta-Analysis (PRISMA-P) 2009 statement checklist (26). Published articles were searched from different major international databases (PubMed, Cochrane Library, Scopus, Web of Science, Science Direct, Google Scholar), and direct Google hand searches were used for additional sources mainly for gray and preprint studies. The Population, Exposure, Comparison and Outcome (PECO) search formula was used to retrieve the articles. All original published and unpublished studies done to determine the prevalence of CCa screening utilization in Ethiopia were extracted to be included in this study. The outcome of interest was the pooled prevalence of uptake of CCa screening among female healthcare professionals. For each of the selected components of PECO, electronic databases were searched using the key words search and the medical subject heading (MeSH) terms. The keywords include “uptake, utilization, cervical cancer, screening, healthcare professional and Ethiopia”. The search terms were combined by the Boolean operators “OR” and “AND”. The specific searching detail in PubMed was putted as ((cervical cancer screening) AND (practice)) OR (utilization)) OR (uptake)) OR (experience)) OR (exposure)) AND (healthcare provider)) OR (healthcare worker)) OR (healthcare professional)) OR (healthcare personnel)) OR (health worker)) AND (Ethiopia)).

### Eligibility criteria and study selection

This review included studies that reported either the use of CCa screening or CCa screening among female health care professionals in Ethiopia. All published and unpublished studies conducted in female health care professionals through first May, 2024 and reported in English language were retrieved to assess eligibility for inclusion in this review. However, this review excluded studies that were case reports, surveillance data (demographic health survey), and abstracts of conferences, articles without full access and the main outcome of interest not reported. The article selection underwent several steps. Two reviewers evaluated the retrieved articles for inclusion using their title, abstract and full text review. Disagreement during the selection process between the reviewers were resolved by consensus after detail discussion. Full texts of selected articles were then evaluated using the eligibility criteria. During the encounter of duplication, only the full-text article was retained.

### Quality assessment and data collection

The Newcastle-Ottawa Scale (NOS) quality assessment tool was used to assess the quality of the included studies. The tool contains three components; selection of the study groups, comparability of the study groups, and ascertainment of exposure or outcome (27). The tool's core component, which was rated on a five-star scale, focused mostly on each primary study's methodological quality. The tool's other component, which was rated between two stars, focused mostly on how comparable each study was. The tool's final component, which was rated from three stars, was used to assess each original study's statistical analysis and findings. Three category criteria totaling a maximum of nine points were included in the NOS. Each study's quality was evaluated using one of the following scoring algorithms: studies with a score of greater than or equal to seven were deemed “good,” those with a score of four to six were deemed “moderate,” and those with a score of less than or equal to three were deemed “poor.” This systematic review result is more valid now because only primary studies of medium to good quality have been included. Using a defined data extraction format, the two reviewers (MA and AW) independently evaluated the publications for overall study quality and extracted data. Primary author, publication year, study region, sample size, prevalence, and the chosen predictors of cervical cancer screening use were all included in the data extraction format.

### Publication bias and statistical analysis

Using the Egger's (28) and Begg's (29) tests with a *p*-value of less than 0.05, the publication bias was evaluated. The heterogeneity between studies was evaluated using the I^2^ statistic and there was no heterogeneity within the included articles. Microsoft Excel was used to extract the data, which were then exported to Stata version 11 for analysis.

## Results

### Study identification and characteristics of included studies

**Figure 1:**
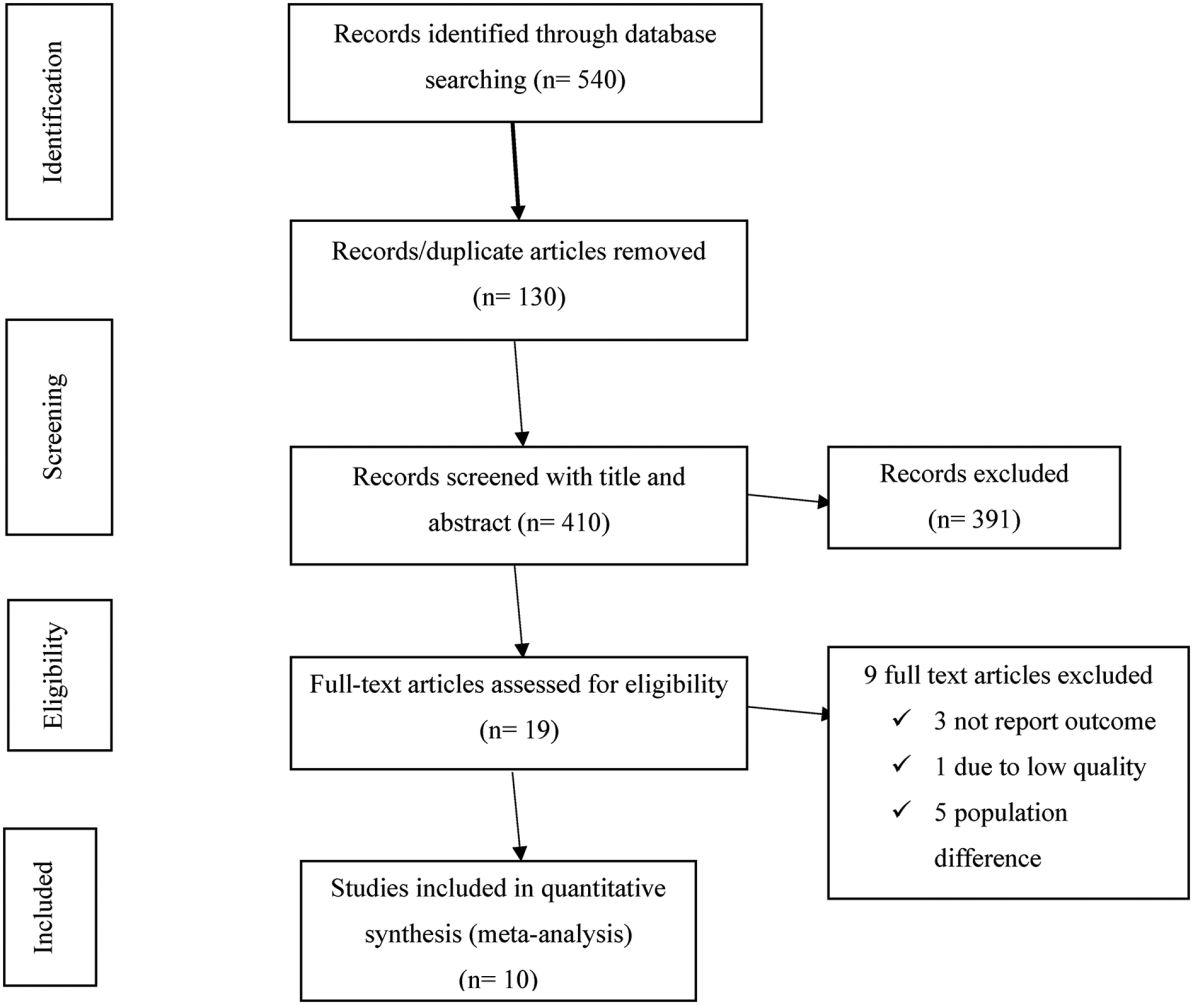
PRISMA flow diagram of cervical cancer screening uptake in Ethiopia.

Both published and unpublished research on the usage of CCa screening among female health care professionals in Ethiopia were included in this systematic review and meta-analysis. A total of 540 research articles were found throughout the assessment procedure. After thorough assessment 410 publications were checked based on their titles and abstracts and 130 duplicate data were removed. After that, the eligibility of 19 full-text papers was assessed based on the inclusion and exclusion criteria. Consequently, three studies were excluded because they failed to provide the expected results (14, 30, 31), one due to low quality (32), and five because the research population was different (16, 33–36). Ten studies were included in the final quantitative meta-analysis (Figure 1).

### Characteristics of the included studies

All the studies included in this review were cross-sectional, and all ten studies were facility-based cross-sectional studies (FBCS). In order to calculate the pooled prevalence of CCa screening, a total of 10 original articles which comprise 2, 919 female health care professionals were included. All the Original articles included in this study were published from 2015 to last May 2024 despite there were no publication year limitation during filtering related studies. From the studies included in this review, the largest sample size was 442 healthcare professionals (37), while the smallest sample size was 164 from a study conducted in other area of Amhara (38).

The studies were distributed as follows: two were conducted in Addis Ababa, three in Amhara, one each in Oromia, Tigray, Sidama, Central Ethiopia region, and one in three different areas of Ethiopia (Addis Ababa, Adama and Bahir Dar). From the total articles included in this review 70% of them were applied simple random sampling (SRS) technique (Table 1).

**Table 1:** Characteristics of studies included in meta-analysis, Ethiopia.

Authors	Publication year	Study area	CCa screening practice (%)	Design	Sample size	Sampling technique
Gebrie et al. (39)	2015	Addis Ababa	8.5	FBCS	275	SRS
Tesfaye et al. (40)	2024	Addis Ababa	18.5	FBCS	293	SRS
Aytenew et al. (38)	2024	Amhara	28.1	FBCS	164	SRS
Kress et al. (41)	2015	Ethiopia	17	FBCS	217	Purposive
Jemal et al. (42)	2023	Central Ethiopia	19.6	FBCS	241	SRS
Gebreegziabher et al (43)	2016	Tigray	10.7	FBCS	242	Purposive
Abebaw et al. (37)	2022	Amhara	8.7	FBCS	442	SRS
Melese et al. (44)	2023	Oromia	14.7	FBCS	266	SRS
Dulla et al. (45)	2017	Sidama	11.4	FBCS	367	SRS
Amare et al. (46)	2022	Amhara	14	FBCS	412	Purposive

FBCS, facility-based cross-sectional; SRS, simple random sampling.

### Meta-analysis of cervical cancer screening uptake in Ethiopia

**Figure 2:**
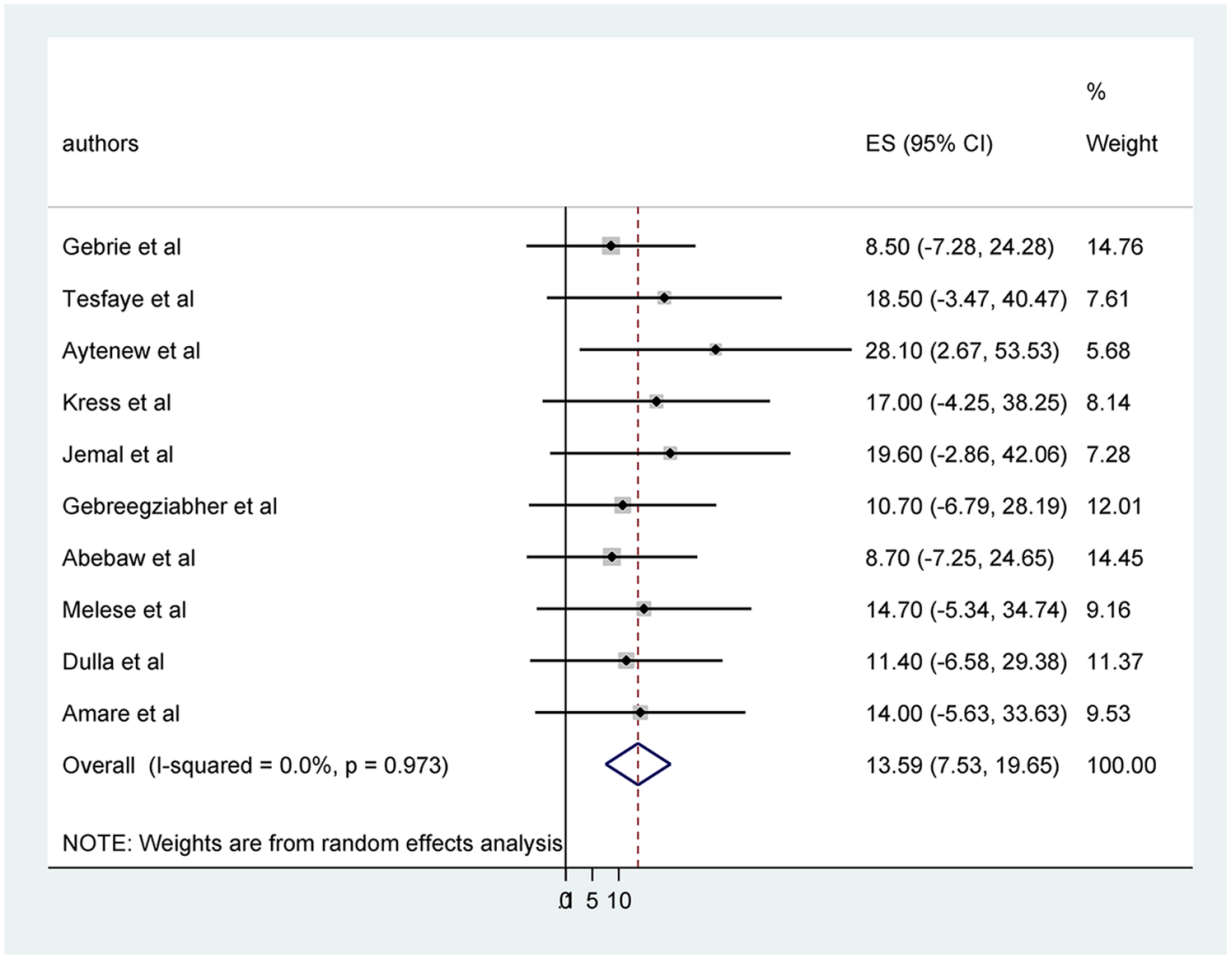
Pooled prevalence cervical cancer screening utilization among healthcare professionals in Ethiopia.

The highest CCa screening uptake was seen in South Gondar hospitals in Amhara region at 28.1% (38). In contrast, the lowest was 8.5% in a study done at Addis Ababa (39) and 8.7% in a study done in the Amhara region (37). The aggregated national level of CCa screening utilization, as determined by a meta-analysis of ten articles, was 13.59% (95% CI: 7.53, 19.65) (Figure 2).

**Figure 3:**
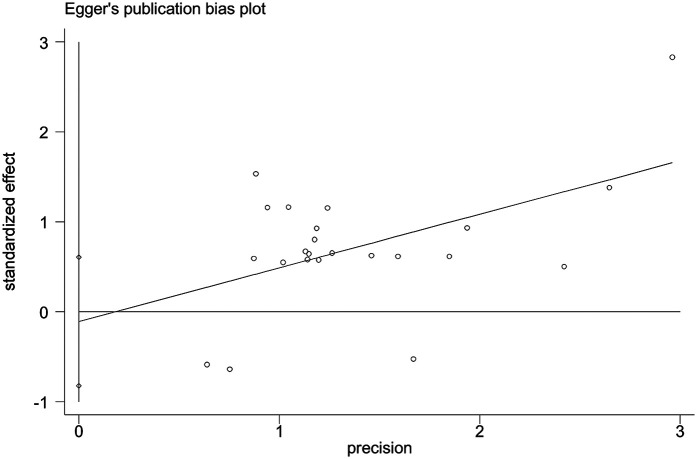
Publication bias analysis using Egger's publication bias plot.

**Figure 4:**
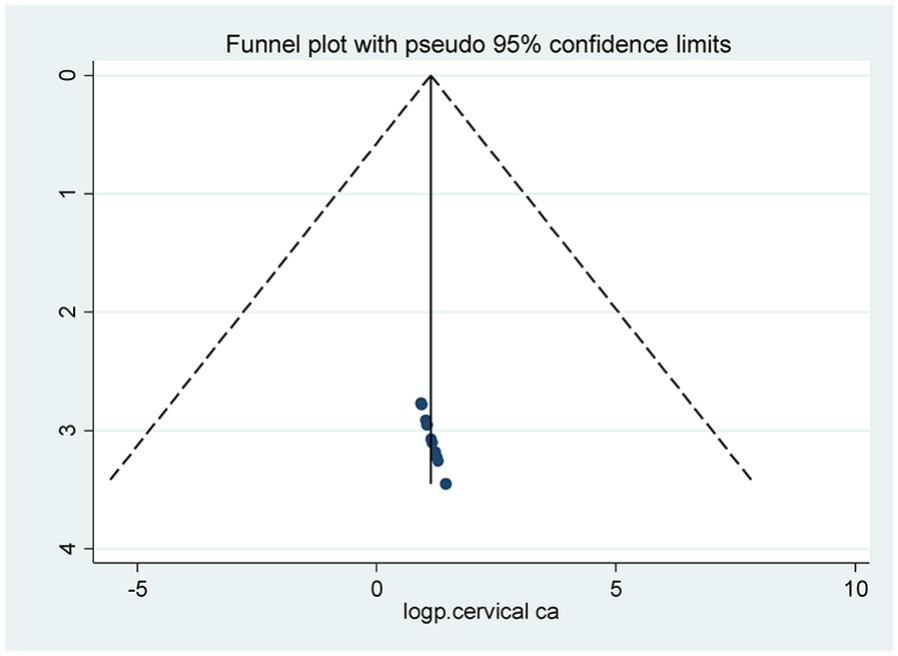
Publication bias by prevalence of cervical cancer screening practice.

Publication bias was assessed using egger's test (Figure 3) and Funnel plot by prevalence of cervical cancer screening practice shows symmetrical distribution (Figure 4).

## Discussion

In Ethiopia, the use of CCa screening programs is not widely established. Nevertheless, the WHO advises that screening tests for CCa be a part of carefully thought out and executed initiatives in every nation's health care strategy. This systematic review aimed to provide a comprehensive estimate of the pooled prevalence of CCa screening uptake among female healthcare professionals in Ethiopia. Consequently, 13.59% (95% CI: 7.53, 19.65) was the pooled national level of CCa screening usage.

The result of the review was consistent with 12.87% in Sub-Saharan Africa (47), 12.70% in India (48), 17% in Nepal (49),19.4% in Kenya (50), 13.46%, 8.11%, and 14.79% in Ethiopia (6, 51, 52). The review results, indicating a prevalence of CCa screening utilization among female healthcare professionals, align with several regional studies, suggesting a consistent trend across various developing regions.

The current review result was lower than 41.0% in Africa (53), 21.4% in China (54), 26.2% in Saudi Arabia (55), and 24.2% in Nigeria (56). This disparity might be due to the demographic characteristics, study settings, and the quality of medical services and screening initiatives. The possible reason might also be the low-risk perception of the healthcare professionals and fear of positive results. The explanation for this could be healthcare professionals not to have been trained to conduct CCa screening were less likely to have positive attitude towards CCa prevention activities (57).

The findings of this literature review also showed that highest CCa screening uptake was seen in South Gondar hospitals in Amhara region and the lowest was in study done at Addis Ababa. The reason might be the difference in the period of study. As the study period difference increases, the interplay of different factors also increase and the practice too (38, 39). Regional variation in the burden of CCa screening in Ethiopia might be explained by the difference in maternal health care service utilization that could be explained by in the difference in spousal support, cultural and linguistic diversity across the regions and societal stigmatization. Additionally, attitude of health care staff may vary by region or other factors may contribute to the difference such as age, educational level, etc. Furthermore, Ethiopia presents varied picture with prevalence across different studies. These variations within the same country might reflect regional disparities, differences in study methodologies, or temporal changes in factors influencing prevalence of practice.

Overall, the review's findings show both parallels and discrepancies with the stated prevalence in a number of comparable regions. These differences highlight how crucial it is to take local factors and context into account when tackling health issues. Furthermore, they contend that although there may be broad patterns, localized factors have a major influence on prevalence rates and ought to be the center of customized interventions and policies.

## Limitation of the review

This systematic review and meta-analysis on CCa screening utilization among female healthcare professionals in Ethiopia has its own limitations. Firstly, it includes only ten studies from six regions, which may affect the representativeness of the findings and limit the generalizability of the conclusions to a national level. The absence of studies from other regions leaves potential regional disparities unaddressed. Secondly, the review relies exclusively on quantitative data, without incorporating qualitative studies that could offer deeper insights into the factors facilitating or hindering CCa screening. As a result, while the review provides valuable quantitative estimates, the lack of qualitative analysis restricts a comprehensive understanding of the underlying factors influencing screening behaviors. These limitations underscore the need for further research to provide more representative and holistic insights.

## Conclusion and recommendations

Cervical cancer is the leading cause of cancer deaths in women in the developing world. Despite, new technologies have been developed to allow for more rapid, cost-effective, and sensitive cervical cancer screening, the utilization of these technologies and services remain minimum. The cervical cancer screening rate was determined to be lower than the WHO recommendations based on this literature review. The low utilization of CCa screening among female healthcare professionals is a significant concern that can impact the broader efforts to combat cervical cancer. Despite their medical knowledge and access to healthcare resources, many healthcare professionals are not engaging in regular screening practices. This underutilization not only jeopardizes the health of the healthcare professionals but also sets a poor example for patients and the community, potentially undermining public health campaigns aimed at increasing screening rates. Based on the this reviews the authors recommend regularly monitor the screening rates among healthcare professionals and evaluate the effectiveness of implemented interventions. Conducting qualitative study to find out underlying reasons for deigning interventions in order to improve utilization and adjust interventions accordingly is also crucial. Through the identification and synthesis of findings from accessible primary studies, this study offers evidence to support policy and program activities and also help the stakeholders and policy makers to consider holistic approaches to address different segments of the eligible population.

## Data Availability

The original contributions presented in the study are included in the article/Supplementary Material, further inquiries can be directed to the corresponding author/s.

## References

[1] Dea S. Estimates of the global burden of cervical cancer associated with HIV. Lancet. (2020) 9. 10.1016/S2214-109X(20)30459-9 PMC7815633 33212031

[2] MoH. F. Federal democratic republic of Ethiopia (FDRE) ministry of health (MoH). Guideline for cervical cancer prevention and control in Ethiopia. (2015).

[3] WHO. World Health Organization. Cancer Ethiopia 2020 country profile. (2020). Available online at: https://www.who.int/publications/m/item/cancer-eth-2020 (Accessed January 01, 2020).

[4] Zhang S Batur P. Human papillomavirus in 2019: an update on cervical cancer prevention and screening guidelines. Clevel Clin J Med. (2019) 86(3):173. 10.3949/ccjm.86a.18018 30849035

[5] Lemp JM De Neve J-W Bussmann H Chen S Manne-Goehler J Theilmann M Lifetime prevalence of cervical cancer screening in 55 low-and middle-income countries. JAMA. (2020) 324(15):1532–42. 10.1001/jama.2020.16244 33079153 PMC7576410

[6] Desta M Getaneh T Yeserah B Worku Y Eshete T Birhanu MY Cervical cancer screening utilization and predictors among eligible women in Ethiopia: a systematic review and meta-analysis. PLoS One. (2021) 16(11):e0259339. 10.1371/journal.pone.0259339 34735507 PMC8568159

[7] Gelibo T Roets L Getachew T Bekele A. Coverage and factors associated with cervical cancer screening: results from a population-based WHO steps study in Ethiopia. Adv Oncol Res Treat. (2017) 1:2.

[8] WHO. WHO guidelines for the use of Thermal Ablation for Cervical pre-cancer lesions. Geneva: World Health Organization (2019). 31661202

[9] FMoH. Federal Ministry of Health E. National Cancer Control Plan of Ethiopia 2016–2020. Vol. 2. Ethiopia: The Minster, Federal Ministry of Health of Ethiopia (2025). Available online at: https://www.iccp-portal.org/sites/default/files/plans/NCCP%20Ethiopia%20Final%20261015.pdf

[10] Shiferaw N Salvador-Davila G Kassahun K Brooks MI Weldegebreal T Tilahun Y The single-visit approach as a cervical cancer prevention strategy among women with HIV in Ethiopia: successes and lessons learned. Glob Health Sci Pract. (2016) 4(1):87–98. 10.9745/GHSP-D-15-00325 27016546 PMC4807751

[11] Kasim J Kalu A Kamara B Alema HB. Cervical cancer screening service utilization and associated factors among women in the shabadino district, southern Ethiopia. J Cancer Epidemiol. (2020) 2020(1):6398394. 10.1155/2020/6398394 32695167 PMC7354647

[12] Nigussie T Admassu B Nigussie A. Cervical cancer screening service utilization and associated factors among age-eligible women in jimma town using health belief model, south west Ethiopia. BMC Women’s Health. (2019) 19:1–0. 10.1186/s12905-019-0826-y 31660938 PMC6819648

[13] Muluneh BA AD Wassie B. Predictors of cervical cancer screening service utilization among commercial sex workers in northwest Ethiopia: a case-control study. BMC Women’s Health. (2019) 19:1–9. 10.1186/s12905-018-0705-y 31842845 PMC6915973

[14] Teame H Gebremariam L Kahsay T Berhe K Gebreheat G Gebremariam G. Factors affecting utilization of cervical cancer screening services among women attending public hospitals in tigray region, Ethiopia, 2018; case control study. PLoS One. (2019) 14(3):e0213546. 10.1371/journal.pone.0213546 30870497 PMC6417770

[15] Aynalem BY Anteneh KT Enyew MM. Utilization of cervical cancer screening and associated factors among women in Debremarkos town, Amhara region, Northwest Ethiopia: community based cross-sectional study. PLoS One. (2020) 15(4):e0231307. 10.1371/journal.pone.0231307 32255807 PMC7138328

[16] Belay Y Dheresa M Sema A Desalew A Assefa N. Cervical cancer screening utilization and associated factors among women aged 30 to 49 years in Dire Dawa, Eastern Ethiopia. Cancer Control. (2020) 27(1):1073274820958701. 10.1177/1073274820958701 33034204 PMC7791449

[17] Idowu A Olowookere SA Fagbemi AT Ogunlaja OA. Determinants of cervical cancer screening uptake among women in Ilorin, north central Nigeria: a community-based study. J Cancer Epidemiol. (2016) 2016. 10.1155/2016/6469240 26880916 PMC4736774

[18] Mugassa AM Frumence G. Factors influencing the uptake of cervical cancer screening services in Tanzania: a health system perspective from national and district levels. Nurs Open. (2020) 7(1):345–54. 10.1002/nop2.395 31871719 PMC6917965

[19] Tigeneh W Molla A Abreha A Assefa M. Pattern of cancer in Tikur Anbessa specialized hospital oncology center in Ethiopia from 1998 to 2010. Int J Cancer Res Mol Mech. (2015) 1(1):1. 10.16966/2381-3318.103

[20] Bray F Ferlay J Soerjomataram I Siegel RL Torre LA Jemal A. Global cancer statistics 2018: GLOBOCAN estimates of incidence and mortality worldwide for 36 cancers in 185 countries. CA:Cancer J Clin. (2018) 68(6):394–424. 10.3322/caac.21492 30207593

[21] Ncube B Bey A Knight J Bessler P Jolly PE. Factors associated with the uptake of cervical cancer screening among women in Portland, Jamaica. N Am J Med Sci. (2015) 7(3):104. 10.4103/1947-2714.153922 25839002 PMC4382764

[22] Stelzle D Tanaka LF Lee KK Khalil AI Baussano I Shah AS Estimates of the global burden of cervical cancer associated with HIV. Lancet Glob Health. (2021) 9(2):e161–e9. 10.1016/S2214-109X(20)30459-9 33212031 PMC7815633

[23] Bedell SL Goldstein LS Goldstein AR Goldstein AT. Cervical cancer screening: past, present, and future. Sex Med Rev. (2020) 8(1):28–37. 10.1016/j.sxmr.2019.09.005 31791846

[24] Juneja A Sehgal A Mitra AB Pandey A. A survey on risk factors associated with cervical cancer. Indian J Cancer. (2013) 40(1):15–22. https://www.researchgate.net/publication/8925144 14716127

[25] Kashyap N Krishan N Kaur S Ghai S. Risk factors of cervical cancer: a case-control study. Asia-Pac J Oncol Nurs. (2019) 6(3):308–14. 10.4103/apjon.apjon_73_18 31259228 PMC6518992

[26] Moher D Shamseer L Clarke M Ghersi D Liberati A Petticrew M Preferred reporting items for systematic review and meta-analysis protocols (PRISMA-P) 2015 statement. Syst Rev. (2015) 4(1). 10.1186/2046-4053-4-1 PMC4320440 25554246

[27] Moskalewicz A Oremus M. No clear choice between NOS and AXIS to assess methodological quality in cross-sectional studies of health-related quality-of-life and breast cancer. J Clin Epidemiol. (2020) 120. 10.1016/j.jclinepi.2019.12.013 31866469

[28] Egger M Smith GD Schneider M Minder C. Bias in meta-analysis detected by a simple, graphical test. Br Med J. (1997) 315(7109):629–34. 10.1136/bmj.315.7109.629 9310563 PMC2127453

[29] Begg CB Mazumdar M. Operating characteristics of a rank correlation test for publication bias. Biometrics. (1994) 50:1088–101. 10.2307/2533446 7786990

[30] Tarekegn AA Mengistu MY Mirach TH. Health professionals’ willingness to pay and associated factors for cervical cancer screening program at College of Medicine and Health Sciences, University of Gondar, Northwest Ethiopia. PLoS One. (2019) 14(4):e0215904. 10.1371/journal.pone.0215904 31039175 PMC6490889

[31] Tsegaye S. Knowledge, Attitude, Practice of Cervical Cancer Screening and Its Associated Factors among Female Students in Hawassa Universitycollege of Medicine and Health Science Hawassa Ethiopia. Addis Ababa: Addis Ababa University (2015).

[32] Bekela E. Assessment of knowledge and attitude of cervical cancer and screening among primary health care workers of West Wollega Zone, Ethiopia, 2016. unpublished.

[33] Shiferaw S Addissie A Gizaw M Hirpa S Ayele W Getachew S Knowledge about cervical cancer and barriers toward cervical cancer screening among HIV-positive women attending public health centers in Addis Ababa city, Ethiopia. Cancer Med. (2018) 7(3):903–12. 10.1002/cam4.1334 29441700 PMC5852347

[34] Nega AD Woldetsadik MA Gelagay AA. Low uptake of cervical cancer screening among HIV positive women in Gondar University referral hospital, Northwest Ethiopia: cross-sectional study design. BMC Women’s Health. (2018) 18:1–7. 10.1186/s12905-017-0499-3 29879969 PMC5992703

[35] Erku DA Netere AK Mersha AG Abebe SA Mekuria AB Belachew SA. Comprehensive knowledge and uptake of cervical cancer screening is low among women living with HIV/AIDS in northwest Ethiopia. Gynecol Oncol Res Pract. (2017) 4:1–7. 10.1186/s40661-016-0036-3 29276611 PMC5738137

[36] Solomon K Tamire M Kaba M. Predictors of cervical cancer screening practice among HIV positive women attending adult anti-retroviral treatment clinics in Bishoftu town, Ethiopia: the application of a health belief model. BMC Cancer. (2019) 19:1–11. 10.1186/s12885-019-6171-6 31646975 PMC6813043

[37] Abebaw E Tesfa M Gezimu W Bekele F Duguma A. Female healthcare providers’ knowledge, attitude, and practice towards cervical cancer screening and associated factors in public hospitals of Northwest Ethiopia. SAGE Open Med. (2022) 10:20503121221095931. 10.1177/20503121221095931 35600715 PMC9118899

[38] Aytenew TM Kassie YT Kebede SD. Uptake of cervical cancer screening and its barriers using health belief model among health professionals working in public hospitals in South Gondar Zone, Northcentral Ethiopia: multicenter cross-sectional study. Women’s Health Rep. (2024) 5(1):152–60. 10.1089/whr.2023.0030 PMC10898230 38414888

[39] Gebrie MH. Knowledge, preventive practice and associated factors of female nurses? Towards cervical cancer in the selected government hospitals in Addis Ababa, Ethiopia. J Diabetes Metab. (2015) 06(07). 10.4172/2155-6156.1000569

[40] Tesfaye W Ashine B Yimer Y Yismaw Y Bitew G Asefa T Utilization of cervical cancer screening and determinant factors among female nurses in selected public hospitals in Addis Ababa, Ethiopia. Cancer Treat Res Commun. (2024) 40:100815. 10.1016/j.ctarc.2024.100815 38733666

[41] Kress CM Sharling L Owen-Smith AA Desalegn D Blumberg HM Goedken J. Knowledge, attitudes, and practices regarding cervical cancer and screening among Ethiopian health care workers. Int J Womens Health. (2015) 7:765–72. 10.2147/IJWH.S85138 26261427 PMC4527576

[42] Jemal Z Chea N Hasen H Tesfaye T Abera N. Cervical cancer screening utilization and associated factors among female health workers in public health facilities of Hossana town, southern Ethiopia: a mixed method approach. PLoS One. (2023) 18(5):e0286262. 10.1371/journal.pone.0286262 37252937 PMC10228814

[43] Gebreegziabher M Asefa NG Berhe S. Factors affecting the practices of cervical cancer screening among female nurses at public health institutions in Mekelle Town, Northern Ethiopia, 2014: a cross-sectional study. J Cancer Research. (2016) 2016:1–7. 10.1155/2016/4743075

[44] Melese A Bekele G Molla E Banga D Agena A Loha A Utilization of cervical cancer screening service among female health workforces in public health institutions in south east Ethiopia, a cross-sectional study. Heliyon. (2024) 10(1):e23086. 10.1016/j.heliyon.2023.e23086 38223710 PMC10784137

[45] Dulla D Daka D Wakgari N. Knowledge about cervical cancer screening and its practice among female health care workers in southern Ethiopia: a cross-sectional study. Int J Womens Health. (2017) 9:365–72. 10.2147/IJWH.S132202 28579837 PMC5446960

[46] Amare YK Bayu NH Worku WZ Gebrie MH Tsega AM Kassa TM Knowledge, preventive practice and associated factors about cervical cancer among female nurses working in West Amhara referral hospitals, Ethiopia. Am J Nurs Scie. (2022) 11(6):163–73. 10.11648/j.ajns.20221106.11

[47] Yimer NB Mohammed MA Solomon K Tadese M Grutzmacher S Meikena HK Cervical cancer screening uptake in Sub-Saharan Africa: a systematic review and meta-analysis. Public Health. (2021) 195:105–11. 10.1016/j.puhe.2021.04.014 34082174

[48] Chawla B Taneja N Awasthi AA Kaur KN Janardhanan R. Knowledge, attitude, and practice on screening toward cervical cancer among health professionals in India—a review. Women’s Health. (2021) 17:17455065211017066. 10.1177/17455065211017066 PMC8371743 34396854

[49] Shrestha A Andersen J Gyawali B Shrestha A Shrestha S Neupane D Cervical cancer screening utilization, and associated factors, in Nepal: a systematic review and meta-analysis. Public Health. (2022) 210:16–25. 10.1016/j.puhe.2022.06.007 35863158

[50] Tiruneh FN Chuang K-Y Ntenda PAM Chuang Y-C. Individual-level and community-level determinants of cervical cancer screening among Kenyan women: a multilevel analysis of a nationwide survey. BMC Women’s Health. (2017) 17:1–14. 10.1186/s12905-017-0469-9 29141612 PMC5688646

[51] Ayenew AA Zewdu BF Nigussie AA. Uptake of cervical cancer screening service and associated factors among age-eligible women in Ethiopia: systematic review and meta-analysis. Infect Agents Cancer. (2020) 15:1–17. 10.1186/s13027-020-00334-3 PMC7666476 33292388

[52] Kassie AM Abate BB Kassaw MW Aragie TG Geleta BA Shiferaw WS. Impact of knowledge and attitude on the utilization rate of cervical cancer screening tests among Ethiopian women: a systematic review and meta-analysis. PLoS One. (2020) 15(12):e0239927. 10.1371/journal.pone.0239927 33290426 PMC7723289

[53] Bogale AL Teklehaymanot T Haidar Ali J Kassie GM. Knowledge, attitude and practice of cervical cancer screening among women infected with HIV in Africa: systematic review and meta-analysis. PLoS One. (2021) 16(4):e0249960. 10.1371/journal.pone.0249960 33831128 PMC8031808

[54] Bao H Zhang L Wang L Zhang M Zhao Z Fang L Significant variations in the cervical cancer screening rate in China by individual-level and geographical measures of socioeconomic status: a multilevel model analysis of a nationally representative survey dataset. Cancer Med. (2018) 7(5):2089–100. 10.1002/cam4.1321 29573569 PMC5943548

[55] Heena H Durrani S AlFayyad I Riaz M Tabasim R Parvez G Knowledge, attitudes, and practices towards cervical cancer and screening amongst female healthcare professionals: a cross-sectional study. J Oncol. (2019) 2019. 10.1155/2019/5423130 31772579 PMC6854973

[56] Owolabi BA Adejumo PO. Utilization of cervical cancer screening service among nurses in ekiti state, Nigeria. Cancer Res J. (2021) 9(1). 10.11648/j.crj.20210901.19

[57] Obol JH LS Obwolo MJ Harrison R Richmond R. Knowledge, attitudes, and practice of cervical cancer prevention among health workers in rural health centres of northern Uganda. BMC Cancer. (2021) 21:1–5. 10.1186/s12885-020-07763-8 33535977 PMC7860193

